# Faith Cure

**Published:** 1886-08

**Authors:** 


### *FAITH CURE.*

[We extract the following from an excellent article on *faith cure* by Romaine J. Curtiss, M.D., as published in the *American Medical Journal*:]

Theological or miraculous cures, as literary curiosities, are as old, or older, than literature; they are as old as tradition. Each of the "Ten Great Religions" appears, from its history, to rest on a foundation of miracle-vending, which is more or less a record of "faith cure." Even Joe Smith performed miracles of this kind, and the Mormon faithful claim that the divine Joe actually raised the dead. In fact, it is a matter of tradition and history, and contemporary observation, that any god,, or any special disciple or representative in the flesh of any god, or even any spiritualist, or any praying girl can perform faith cures. We have shrines for faith cure, as the holy well at Lourdes, which is advertised as the medicine of faith cure which is worked by "the Virgin." There is the holy well at Mecca, which has performed miracle cures for centuries for the Mahommedans, though the water is more filthy than London sewage.

But I mention these things to show that faith cure is not a distinctive power of any god, or any religion, or any faith, it is simply a faith in almost anything that is supernatural, in the first place; and, in the second place, it appears to make no possible difference in a faith cure whether the object of faith is even supernatural. Faith in a dried potato, or an old chestnut, or a mad-stone, or the bones of an old saint, or a snake's tooth, or alligator's tusk, or the false tongue of a colt, or the dung of serpents, or Joss paper, and a thousand other things, will have just as good effects in working faith cures as the power of Jehovah; and, so far as the clergy are concerned, it is about an even thing, relating to their testimony, whether faith or patent medicines have the lead in working the cure of disease.

These facts are all correlatives of the conception which now prevails among thinking people—which means people who can think—that the Divine energy, or Almighty God, does not run this universe by special exhibitions of power—not even to cure a woman's headache. In the first place, if we admit or claim the contrary, then we claim or admit that the power of Almighty God can do no greater things than a serpent's dung, or the most degraded deity of the Fiji Islands.

God does not work in any such way. The whole nation, and, in fact, the whole of the nations, united in the praying for the cure of Garfield, without effect. Is it God-like to reject the plea of the whole human family and recognize that of a hysterical woman; or is such conduct simply capricious? But in the second place, unless we deny that God works by special miraculous power, we deny that he is God, for such a method implies that God has made a mistake. It is virtually an admittance and confession of error and a mistake on the part of the Deity to work a miracle, whether the miracle is a faith cure or anything else. Of course, God lays his plans, and works by law or by method, and our conception cannot acknowledge that God can make a mistake, either in his plans or work, and consequently a "special interposition" is never required, and, if ever indulged in, destroys the reputation of the Deity for infinite wisdom and power.

An acquaintance of writer, a married lady, had consumption, and died about a year ago from this disease. She lived near a city where there is an institution for faith cure. One of the praying girls was sent to see this lady, who was then in the last stage of the disease. After a day or two of intermittent praying, the invalid declared herself cured. The friends and neighborhood were startled by a reported miracle in their midst. The lady got out of bed, and resumed the care of her house. The doctor was confounded by the cessation of her night sweats, cough and expectoration. She took food—eating, in fact, ravenously. Matters went on this way, with frequent meetings of church perple for "praise," for two weeks, when the woman suddenly died from strangulation.

The explanation of the whole matter is very simple. The prayers gave her a dominant idea, which controlled her nerves of sensation and motion. She felt no pain, and, as she could feel nothing of the accumulating matter in her lungs, she had no cough, and the lungs gradually filled, until there was no lung-room left; when the lungs could contain no more sputa the poor lady died.

